# Does dorsal capsule interposition improve the results of proximal row carpectomy in Kienböck’s disease? One year randomized trial

**DOI:** 10.1051/sicotj/2015026

**Published:** 2015-09-22

**Authors:** Walter Yoshinori Fukushima, Vinícius Ynoe de Moraes, Fernado Travaglini Penteado, Flávio Faloppa, João Baptista Gomes dos Santos

**Affiliations:** 1 Universidade Federal de São Paulo (UNIFESP), Department of Orthopaedics and Traumatology SP Brazil

**Keywords:** Kienböck’s disease, Carpectomy, Randomized trial, Lunate osteonecrosis

## Abstract

*Introduction*: Proximal row carpectomy (PRC) is an option as a salvage procedure in late stage Kienböck’s disease. In this study, we hypothesize that interposition of a dorsal capsular flap following PRC improves functional outcomes. No comparative study is available to assess whether interposition is effective from the functional perspective. This study aims to determine whether the addition of this procedure may improve functional outcomes at a one year assessment.

*Methods*: Thirty adult patients with IIIA and IIIB Lichtman stages, aged 18–54 years, were randomized into two study groups. Fourteen patients were allocated to the “no interposition group” and 16 to the “interposition” group. DASH questionnaire was used to evaluate quality of life. Cooney’s system was used to assess pain, functional state, range of motion, and grip strength. Complications were also assessed. Final followup and clinical assessment occurred after 12 months.

*Results*: After 12 months and no patient losses, outcomes were similar in both groups. DASH scores (41.9 (7.5) vs. 42.9 (12.8), *p* = 0.79)), Cooney’s system (poor results, 0.6 vs. 0.14, *p* = 0.54), and complications were similar between groups. In conclusion, the inclusion of a dorsal capsular flap does not improve functional outcomes in PRC. Low rates of complications were found in both groups.

## Introduction

Kienböck’s disease is characterized by idiopathic avascular necrosis of the lunate bone, with increased predominance in young individuals [1]. It often presents with the onset of pain, progressive loss of grip strength, and functional impairment. Osteoarthritis occurs as the condition progresses. These symptoms result in discomfort, reduced working capacity, early retirement, and decreased quality of life [1, 2].

Controversy still remains regarding the best treatment option for late stage disease (Lichtman stages IIIA/IIIB) [3]. Currently, proximal row carpectomy (PRC) is indicated [4] with some series demonstrating good results with this technique [5–7]. PRC has been combined with dorsal flap interposition, radial styloidectomy, and osteotomy of the head of the capitate [8].

In this study, we have hypothesized that the interposition of a flap of the dorsal capsule (covering the head of the capitate) may improve functional outcomes. The purpose of this randomized clinical trial is to evaluate the differences between a proximal row carpectomy with and without interposition of a “U-” shaped, distally based dorsal capsular flap in stage IIIA and IIIB Kienböck’s disease.

## Materials and methods

The present study is a prospective randomized clinical trial including 30 patients. Research methods were approved by the Local Ethics Committee. The study protocol is registered in the ****** Clinical Trials Registry *****.

Patients were randomized into groups with or without capsule interposition by a random generation of numbers that were kept in opaque envelopes. Envelopes were opened on the day of surgery to ensure proper allocation concealment. Data were analyzed according to the intention-to-treat principle.

All of the patients submitted for clinical and radiographic assessment were healthy, active adults. Diagnoses were made by radiographic assessment by two of the senior authors (Fukushima and Dos Santos) with histopathological confirmation after the procedure.

At 12 months, the degree of pain was evaluated according to Cooney’s qualitative scale as “Absent”, “Mild”, “Moderate”, and “Intense” [9, 10]. The DASH questionnaire [11] was also administered. Complications were assessed in the short term (1, 3, and 6 weeks) and at 12 months. Patient outcomes were assessed by researchers not associated with the study design. Lichtman’s modified classification [12] was used to guide treatment and patient inclusion. Patients were included if classified as stage IIIA or IIIB. Return to work time was also assessed and considered as the period between surgery and return to work.

### Surgical technique

**Figure 1.**
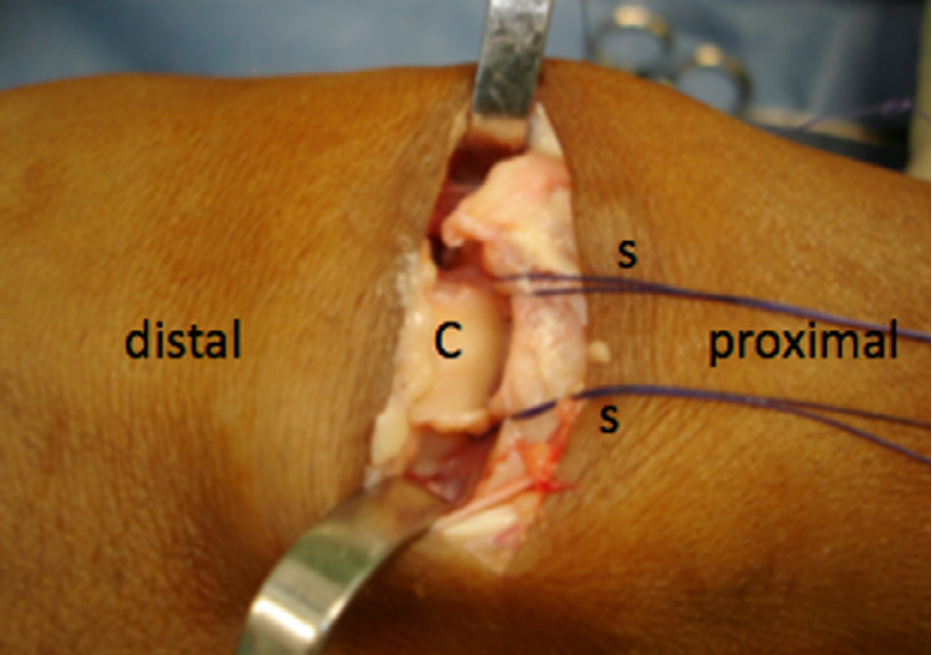
Capitate head exposed and sutures being placed at the volar capsule. S *=* suture lines; C *=* capitate head.

Surgery consisted of proximal row carpectomy, as well as a posterior interosseous neurectomy, and radial styloidectomy. In Group A, interposition of the dorsal capsule was performed between the radius and the capitate following removal of the scaphoid, lunate, and triquetrum bones. A U-shaped distally based dorsal capsular flap was interposed and sutured at the insertion of the volar capsule (Figure 1).

In Group B, there was no capsular interposition and the surgical steps were identical, with exception to the opening of the dorsal capsule that was exposed transversally, without the dorsal U-shaped flap and its interposition. All received the same postoperative protocol: patients used a wrist cast for 3–4 weeks. Therapy is managed by an experienced hand therapist (improvement of range of motion and strength) for 6 weeks.

### Statistical methods

Mann-Whitney test was used for comparison of continuous data, and the Fisher F test was used for categorical data. *P* < 0.05 was considered statistically significant. SPSS 16 for Windows (Statistical Program for the Social Services Inc, Chicago, IL, USA) program was used for statistical analysis.

Sample size calculation considered a 6-point DASH difference (*α* = 0.05 and *β* = 0.80), considering minimal clinically important differences range [13, 14].

## Results

All the 30 included patients were available at the 12-month followup. Median age was 36.5 years, ranging from 18 to 54 years. Twenty of the patients were male and 23 were right-handed. Groups’ demographics were similar (Table 1), ensuring proper randomization.

**Table 1.** Baseline demographics.

Outcome	Interposition	No interposition	*p* value
Age (mean, range)		2 (14)	0.54*
Gender (male, %)	41.9 (7.5)	42.9 (12.8)	0.79**
Side affected (right, %)	5.6 (2.6)	6.8 (2.6)	0.44**
Complications – minor (%)	1 (6)	2 (14)	0.54*

* Fisher F test.

** Mann-Whitney test.

No differences between groups were observed as depicted in Table 2. Three complications were recognized: one sympathetic reflex dystrophy and two patients with superficial incision infection. A dedicated analysis of individual patient outcomes may be found in supplementary material.

**Table 2.** Outcomes – 12 months.

Outcome	Interposition	No interposition	*p* value
Cooney – poor results (%)	1 (6)	2 (14)	0.54*
DASH score – mean (*SD*)	41.9 (7.5)	42.9 (12.8)	0.79**
Return to work – days (*SD*)	5.6 (2.6)	6.8 (2.6)	0.44**
Complications – minor (%)	1 (6)	2 (14)	0.54*

* Fisher F test.

** Mann-Whitney test.

## Discussion

Our data demonstrates no benefit of dorsal capsule interposition in PRC. The interposition of the dorsal capsule may add some benefit as it spares direct contact between the lunate facet and the head of the capitate, however, this did not result in more positive outcomes in this study.

The primary flaws of this Level 1 study relate to the small sample size and relatively brief followup period. Thus we are not able to infer that the use of this interposition acts as a safeguard for wrist osteoarthritis. Small sample sizes are related to type II error, however, no trend favoring interposition was recognized for any of the studied outcomes.

**Figure 2.**
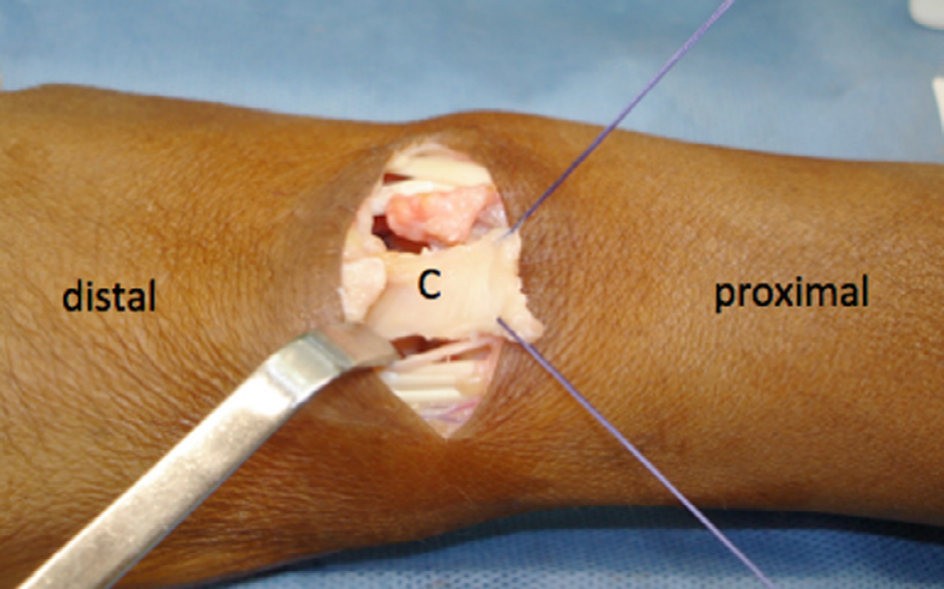
Intraoperative details of the distally based dorsal flap. C *=* capitate head.

We performed a radial styloidectomy at the distal portion of the styloid in all patients due to radiocarpal impact that occurs during radial deviation [15]. The presence of a distally based flap covering the head to the capitate has two theoretical advantages: it spares the contact between the lunate facet and the capitate and stabilizes the capitate, as the capsule is still fixed distally in the bone and in the volar capsule, improving stabilization after PRC (Figure 2).

Controversy exists as to the best operative technique for the treatment of Kienböck’s disease. Proximal row carpectomy showed promising results and permitted some degree of joint motion, improving upper limb function during daily living and working activities [16]. We highlight that intercarpal arthrodesis is also a treatment option, with satisfactory results reported [17–19].

New options such as rib autograft [20] and pyrocarbon implants [21] are available and need further investigation. Despite the many treatment options, an evidence-based approach demonstrates no superiority for any surgical treatment in early or late stage disease and the authors underscore the lack of randomized trials [3], which is unfortunately commonplace in hand surgery research [22, 23].

Dorsal capsule interposition is not effective as an additional step following proximal row carpectomy in Kienböck’s disease. Further studies shall focus on comparing with new treatment options and longer period assessments (five years), specially considering osteoarthrosis as an important outcome.

## Conflict of interest

All the authors have no conflict of interest regarding to the content of this manuscript.

## Supplemental material

**Appendix** – Table A1. Individual results according to each case, surgery performed, clinical results, DASH questionnaire, return to work (in months), and complications.
