# Supplementary material for: Does dorsal capsule interposition improve the results of proximal row carpectomy in Kienböck’s disease? One year randomized trial
Source: SICOT J. 2015 Sep 22;1:25. doi: 10.1051/sicotj/2015026 (PMC4881010; doi:10.1051/sicotj/2015026)
Supplement: Supplementary file 1 — Appendix – Table A1. Individual results according to each case, surgery performed, clinical results, DASH questionnaire, return to work (in months), and complications. [file sicotj150026-sicotj-1-25-s1.pdf]

Appendix

Table – Individual results according to each case, surgery performed, clinical results, DASH questionnaire, return to work (in months) and complications.

Case	Surgery	Clinical results	DASH	Return to work	Complications
1	With	Good	33,7	No	
2	Without	Excellent	43,3	2	
3	With	Excellent	36,5	10	
4	Without	Excellent	37,7	7	
5	With	Excellent	42,1	3	
6	With	Fair	44.9	No	
7	Without	Good	66,2	No	Dystrophy
8	With	Good	53,3	3	
9	With	Good	45,5	5	Infection
10	Without	Excellent	34,9	8	
11	With	Good	43,3	No	
12	With	Good	40,4	No	
13	Without	Good	40,6	2	
14	Without	Excellent	45	11	
15	Without	Excellent	30.4	12	
16	Without	Good	45.0	No	
17	Without	Good	32.5	No	
18	with	Good	44.1	No	
19	with	Good	51.9	5	
20	Without	Fair	60.3	No	Infection
21	Without	Excellent	24.1	7	
22	Without	Fair	34,6	No	
23	with	Good	40.8	No	
24	with	Good	52.5	No	
25	with	Excellent	35.0	6	
26	with	Good	39.9	9	
27	with	Excellent	24.1	4	
28	With	Good	42.5	No	
29	Without	Good	65.0	No	
30	Without	Good	41.1	6	

With=with interposition of the dorsal capsule
Without=without interposition of the dorsal capsule
